# Identification of Therapeutic Targets for Hyperuricemia: Systematic Genome-Wide Mendelian Randomization and Colocalization Analysis

**DOI:** 10.3390/biomedicines13051022

**Published:** 2025-04-23

**Authors:** Na Chen, Leilei Gong, Li Zhang, Yali Li, Yunya Bai, Dan Gao, Lan Zhang

**Affiliations:** 1 Department of Pharmacy, Xuanwu Hospital Capital Medical University, Beijing 100053, China; 2 Beijing Obstetrics and Gynecology Hospital, Capital Medical University, Beijing 100026, China

**Keywords:** hyperuricemia, Mendelian randomization, summary-data-based Mendelian randomization, colocalization, druggable genes

## Abstract

**Background**: At present, there are still limitations and challenges in the treatment of hyperuricemia (HUA). Mendelian randomization (MR) has been widely used to identify new therapeutic targets. Therefore, we conducted a systematic druggable genome-wide MR to explore potential therapeutic targets and drugs for HUA. **Methods**: We integrated druggable genome data; blood, kidney, and intestinal expression quantitative trait loci (eQTLs); and HUA-associated genome-wide association study (GWAS) data to analyze the potential causal relationships between drug target genes and HUA using the MR method. Summary-data-based MR (SMR) analysis and Bayesian colocalization were used to assess causality. In addition, we conducted phenome-wide association studies, protein network construction, and enrichment analysis of significant targets to evaluate their biological functions and potential side effects. Finally, we performed drug prediction and molecular docking to identify potential drugs targeting these genes for HUA treatment. **Results**: Overall, we identified 22 druggable genes significantly associated with HUA through MR, SMR, and colocalization analyses. Among them, two prior druggable genes (*ADORA2B* and *NDUFC2*) reached statistically significant levels in at least two tissues in the blood, kidney, and intestine. Further results from phenome-wide studies revealed that there were no potential side effects of *ADORA2B* or *NDUFC2*. Moreover, we screened 15 potential drugs targeting the 22 druggable genes that could serve as candidates for HUA drug development. **Conclusions**: This study provides genetic evidence supporting the potential benefits of targeting 22 druggable genes for HUA treatment, offering new insights into the development of targeted drugs for HUA.

## 1. Introduction

Hyperuricemia (HUA), characterized by elevated serum uric acid (UA) levels, is the fourth most common metabolic disease after diabetes, hypertension, and hyperlipidemia [1]. The global prevalence of HUA has shown a significant upward trend. Epidemiological studies reveal that approximately 18% of the Chinese population is affected by this condition, while the prevalence rates in Western countries, including the United States, Mexico, and Ireland, are even higher, exceeding 20% [2,3,4,5]. The long-term elevation of UA levels can precipitate the deposition of urate crystals in joints, the kidneys, and other tissues, ultimately contributing to gout, hyperuricemic nephropathy, and metabolic syndrome. In addition, HUA serves as a risk factor for numerous chronic diseases, such as diabetes, hypertension, coronary heart disease, and venous thromboembolism [6,7]. Despite UA’s physiological roles in antioxidant defense, elevated levels are deleterious and require therapeutic intervention. The drugs commonly used for HUA in clinical practice include inhibitors of xanthine oxidase, urate transporter inhibitors, and uricase, among which the representative drugs are allopurinol, benzbromarone, and febuxostat [8]. In addition, novel therapeutic strategies, including the administration of selected intestinal anaerobic purine-degrading bacteria (PDB), the regulation of intestinal urate transport, and the use of liver-targeted xanthine oxidorereductase mRNA knockdown, are emerging but are still under investigation [9]. At present, the clinical treatment of HUA still focuses on UA-lowering drugs, such as allopurinol and febuxostat. However, these medications are burdened with several limitations, including their ability to cause severe allergic reactions, drug interactions, and an increased risk of cardiovascular death [10,11,12,13]. Therefore, there is a pressing need to explore the underlying pathogenesis of HUA and identify novel therapeutic targets.

The incorporation of genetics into the drug development process presents a novel and promising avenue. Large-scale human genetic investigations offer a unique opportunity to advance the development of novel therapeutics for numerous complex diseases, as genetically validated drug targets possess an increased likelihood of success within the drug discovery pipeline [14,15]. Mendelian randomization (MR) is a method that uses genetic variation as an instrumental variable to infer causal relationships between the exposure and outcome and it is often regarded as a natural randomized controlled trial [16]. As an emerging method for causal inference, MR has demonstrated remarkable strengths in the field of drug target identification by integrating aggregated data from disease GWAS and expression quantitative trait locus (eQTL) studies [17,18]. The eQTLs identified within the genomic domains of pharmacologically actionable genes are often regarded as proxies, given that the expression levels of these genes can be interpreted as a form of lifelong exposure [19]. Hence, the MR-based screening of target proteins has robust natural advantages and genetic substantiation and has been employed in the screening of drug targets for diseases such as Alzheimer’s disease [20], sarcopenia [21], migraine [22], amyotrophic lateral sclerosis [23], idiopathic pulmonary fibrosis [24], and chronic kidney disease [25].

In this study, we performed a systematic druggable genome-wide MR analysis to identify therapeutic targets for HUA. First, we obtained data on druggable genes and conducted a meticulous screening process, focusing on genes associated with blood eQTLs, kidney eQTLs, and intestine eQTLs. These selected genes were then subjected to two-sample MR analysis with genome-wide association study (GWAS) data and serum UA levels to identify genes strongly associated with HUA. We subsequently employed summary-data-based MR (SMR) and colocalization analysis to increase the credibility of our findings. Furthermore, protein network construction, enrichment analysis, and phenome-wide association analysis were conducted for significant genes to explore the biological functions of therapeutic targets and their potential clinical associations. Finally, we conducted candidate drug prediction and molecular docking analysis for key genes to identify potential therapeutic drugs for HUA.

## 2. Materials and Methods

**Figure 1:**
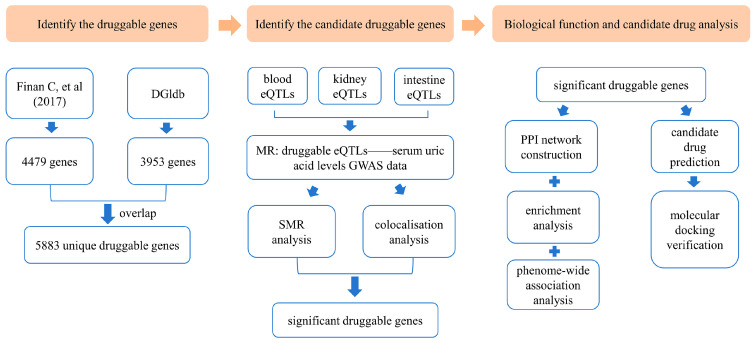
Overview of the study design [26]. DGIdb: Drug–Gene Interaction Database; eQTLs: expression quantitative trait loci; MR: Mendelian randomization; GWAS: genome-wide association study; SMR: summary-data-based Mendelian randomization; PPI: protein–protein interaction.

An overview of this study is illustrated in Figure 1.

### 2.1. Identification of Druggable Genes

The Drug–Gene Interaction Database (DGIdb, https://www.dgidb.org/, accessed on 3 October 2024) [27] and a contemporary research report by Finan, C. et al. [26] served as sources for the identification of druggable genes. The DGIdb compiles and disseminates knowledge on drug–gene interactions and druggable genes, drawing from a broad spectrum of publications, databases, and other web-based resources. We accessed DGIdb’s category data, which were updated in February 2022. Additionally, we incorporated a list of druggable genes compiled in a review conducted by Finan et al. By integrating druggable genes from both sources, we obtained a more comprehensive collection of druggable genes.

### 2.2. Expression Quantitative Trait Locus (eQTL) Datasets

Given that cis-regulatory elements have more direct and specific biological effects than trans-regulatory elements, we utilized cis-eQTLs from human blood, kidneys, and intestine tissue (genetic variants within 1 Mb on either side of the drug-available gene coding sequence). The blood, kidney, and intestine eQTL datasets were all derived from the genotype–tissue expression data (GTEx analysis V8, https://gtexportal.org/home/downloads/adult-gtex/qtl, accessed on 11 October 2024), which included data from 838 donors of European ancestry comprising 17,382 samples across 52 tissues and two cell lines [28]. We selected all statistically significant eQTLs (*p* < 1 × 10^−8^) corresponding to genes with expression levels greater than 0.1 fragments per kilobase per million mapped fragments in at least 10 samples, along with a comprehensive set of single-nucleotide polymorphism (SNP) information. After filtering, we finally acquired 7,614 blood eQTLs, 589 brother kidney eQTLs, and 2888 intestine eQTLs.

### 2.3. Hyperuricemia Genome-Wide Association Studies (GWAS) Dataset

This study utilized GWAS summary statistics derived from a large-scale cohort. The outcome data for HUA represented by serum UA levels (GWAS ID: ebi-a-GCST90018977) comprised genetic data from 343,836 participants of European ancestry with 19,041,286 SNPs [29].

### 2.4. Mendelian Randomization Analysis

The MR analysis was conducted following the STROBE-MR checklist (Supplementary Materials) [30]. The “TwoSampleMR” package (version 0.6.6) was employed to perform MR analysis using R software (version 4.3.1) [31]. The eQTLs of the drug genome were chosen as the instrumental variables (IVs). The selection of eQTLs adhered to the following criteria: (1) only eQTLs demonstrating a significant association with the exposure (*p* < 1 × 10^−8^) were included; (2) to minimize linkage disequilibrium (LD) bias, eQTLs were required to be independent, defined by an r^2^ threshold of <0.001 using European samples from the 1000 Genomes Project and a minimum genetic distance of 10,000 kb; and (3) eQTLs exhibiting associations with potential confounders or outcomes were excluded to ensure specificity. The strength of the IVs was estimated using the F statistic, and a threshold of F > 10 was selected to exclude weak instruments. All palindromic SNPs with a minor allele frequency (MAF) > 0.40 were removed. The LDtrait Tool in LDlink (https://ldlink.nih.gov/?tab=home, accessed on 15 October 2024) was utilized to identify phenotypes associated with the SNPs. After harmonizing the filtered SNPs, MR analyses were performed. The Wald ratio method was used to perform MR analysis when only one SNP was available for analysis, whereas the inverse-variance weighted (IVW) method with random effects was used to conduct MR estimation when multiple SNPs were available. Statistical significance was defined as a false discovery rate (FDR) < 0.05. Moreover, Cochran’s Q test was used to test for heterogeneity among the individual causal effects of SNPs, while MR-Egger was performed to consider horizontal pleiotropy [32,33]. The MR Steiger directionality test was performed to check whether the exposure had a directional causal relationship with the outcome.

### 2.5. Summary-Data-Based Mendelian Randomization Analysis

To further validate the causal relationships between candidate genes and serum UA levels, we conducted an SMR analysis [34]. The Heterogeneity in Dependent Instruments (HEIDI) test, which incorporates multiple SNPs within a specific region, was utilized to distinguish candidate genes whose association with the risk of high serum UA levels stemmed from a common genetic variant rather than genetic linkage. This test incorporates multiple SNPs within a specific region to assess potential confounding by linkage disequilibrium (LD). The SMR and HEIDI tests were performed using the SMR software package (version 1.3.1). The statistical significance thresholds were set as follows: an FDR < 0.05 for SMR analysis and a HEIDI test *p* value > 0.05 to indicate that the observed associations were not driven by LD.

### 2.6. Colocalization Analysis

To evaluate the potential shared causal genetic variations in physical location between serum UA levels and eQTLs, we performed Bayesian colocalization analysis using summary statistics from UA-GWAS and eQTL datasets via the “coloc” package in R [35]. Specifically, SNPs located within ±100 kb from each UA level gene’s TSS were separately filtered from the GWAS data for the serum UA levels, blood eQTL data, kidney eQTL data, and intestine eQTL data. The probability that a given SNP is associated with serum UA levels is denoted P1, the probability that a given SNP is a significant eQTL is denoted P2, and the probability that a given SNP is an outcome of both serum UA levels and eQTLs is denoted P12. The probabilities were set as P1 = 1 × 10^−4^, P2 = 1 × 10^−4^, and P12 = 1 × 10^−5^. The posterior probability (PP) was used to quantify the support for five hypotheses: there was no causal variant for either trait in the genomic locus (PPH0); there was one causal variant associated with gene expression but not serum UA levels (PPH1); there was one causal variant associated with serum UA levels but not gene expression (PPH2); there were two distinct causal variants associated with serum UA levels and gene expression (PPH3); and there was a shared causal variant associated with serum UA levels and gene expression (PPH4). Genes with PPH4 > 0.5 were considered as providing medium support for colocalization, and those with PPH4 > 0.75 were considered strong support for colocalization [36,37].

### 2.7. Phenome-Wide Association Analysis

To investigate potential causal linkages between identified druggable genes and various disease phenotypes while assessing their possible adverse effects, we conducted a phenome-wide association study. The IEU OpenGWAS Project (https://gwas.mrcieu.ac.uk/phewas/, accessed on 5 November 2024) was used to examine a phenome-wide association study of SNPs corresponding to druggable genes that showed significance in MR, SMR, and colocalization analyses [31].

### 2.8. Protein–Protein Interaction Network Construction and Enrichment Analysis

To explore the functional relationships among the significant druggable genes, we constructed a protein–protein interaction (PPI) network tailored specifically for the identified overlapping genes using the Search Tool for the Retrieval of Interacting Genes/Proteins (STRING) database (https://string-db.org, accessed on 9 November 2024) [38]. The network was configured exclusively for “*Homo sapiens*” as the focal species, with an interaction confidence score threshold > 0.4 to ensure high-quality interactions. The resulting PPI network data were then imported into Cytoscape 3.9.1 for visualization [39]. The node importance was determined by the degree algorithm, with the node color and size adjusted according to degree values. Nodes with degree values >10 were considered to have significant interactions.

For the functional characterization of the druggable genes, we performed Gene Ontology (GO) and Kyoto Encyclopedia of Genes and Genomes (KEGG) pathway enrichment analyses using the Database for Annotation, Visualization, and Integrated Discovery (DAVID, https://david.ncifcrf.gov/, accessed on 13 November 2024) database [40]. The GO functional annotations covered three categories: biological process (BP), cellular component (CC), and molecular function (MF). The results were visualized using bar or bubble charts to highlight the most significant functional terms.

### 2.9. Candidate Drug Prediction

To identify potential therapeutic agents targeting the significant druggable genes identified in our study, we performed drug predictive analysis using the Drug Signatures Database (DSigDB, http://dsigdb.tanlab.org/DSigDBv1.0/, accessed on 15 November 2024) [41]. DSigDB is a new gene set resource that relates drugs or compounds and their target genes; it currently holds 22,527 gene sets and consists of 17,389 unique compounds spanning 19,531 genes. The previously identified significant druggable genes were uploaded into DSigDB, and, then, the list of predicted drugs obtained with *p* values < 0.05 was downloaded.

### 2.10. Molecular Docking Verification

To validate the pharmacological potential of candidate drugs, we conducted molecular docking simulations to assess the binding affinities and interaction patterns between the potential drug candidates and their target proteins. First, the two-dimensional (2D) chemical structures of the compounds were retrieved from the PubChem website. Each compound was configured in AutoDock Vina 1.1.2 through eliminating water molecules, appending hydrogen atoms, and designating the drug as the ligand, following established protocols [42]. Subsequently, the torsion tree was automatically configured by the software, and the resulting configuration was exported as a PDBQT-formatted ligand file. For protein preparation, the crystal structures of the target proteins were obtained from the Protein Data Bank (PDB) database (http://www.rcsb.org/, accessed on 27 November 2024) [43]. Prior to docking, these structures underwent a preprocessing step, which involved the removal of redundant protein chains, ligands, and water molecules and the addition of hydrogen atoms to optimize compatibility with the docking software. The docking simulations between compounds and target proteins were subsequently performed using AutoDock Vina 1.1.2. Finally, the three-dimensional (3D) structures of the molecular ligand–protein receptor complexes were visually represented using PyMol software (version 2.5.0). The affinity and strength of the molecular docking interactions were quantified based on binding energy.

## 3. Results

### 3.1. Druggable Genome

By querying the DGIdb v4.2.0, we identified a total of 3953 potential druggable genes (Table S1). Additionally, we extracted 4479 druggable genes from a previous review (Table S2). Following data integration, a final set of 5883 unique druggable genes, annotated according to the nomenclature guidelines of the Human Genome Organization Gene Nomenclature Committee, was compiled for subsequent analysis (Table S3).

### 3.2. Candidate Druggable Genes

**Figure 2:**
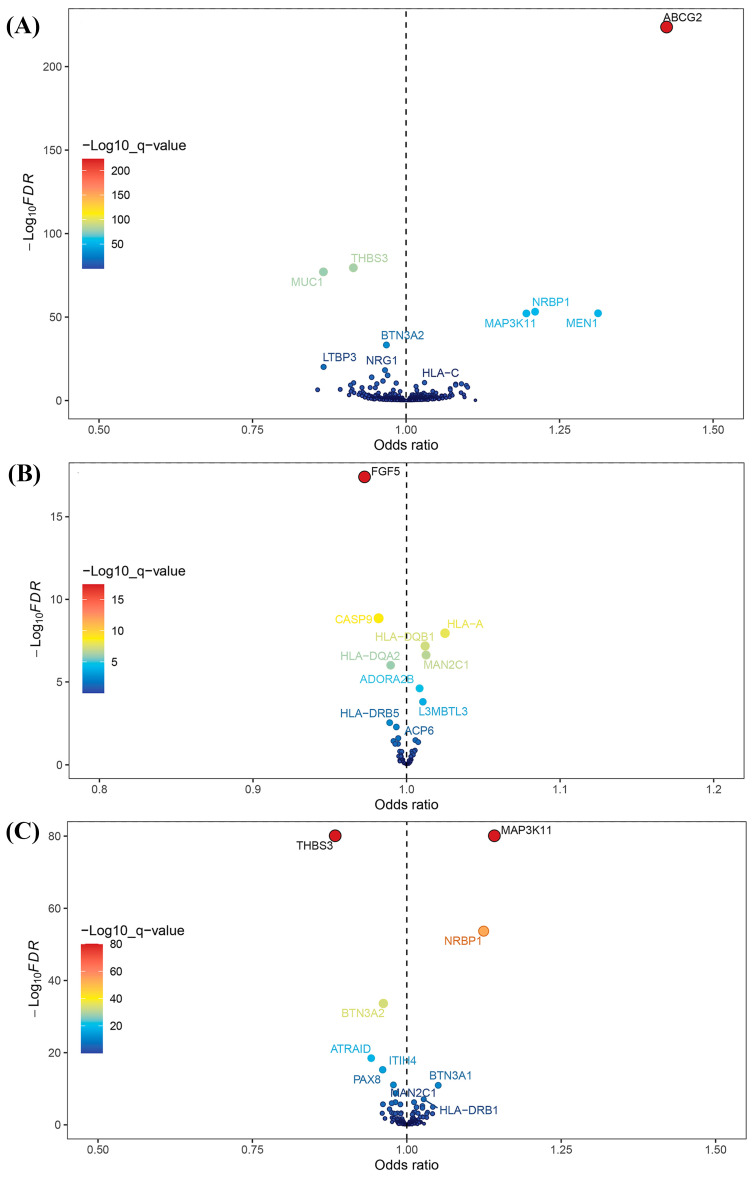
Volcano plots for Mendelian randomization (MR) results. (**A**) Volcano plot for MR results between blood eQTLs and serum uric acid levels. (**B**) Volcano plot for MR results between kidney eQTLs and serum uric acid levels. (**C**) Volcano plot for MR results between intestine eQTLs and serum uric acid levels.

**Figure 3:**
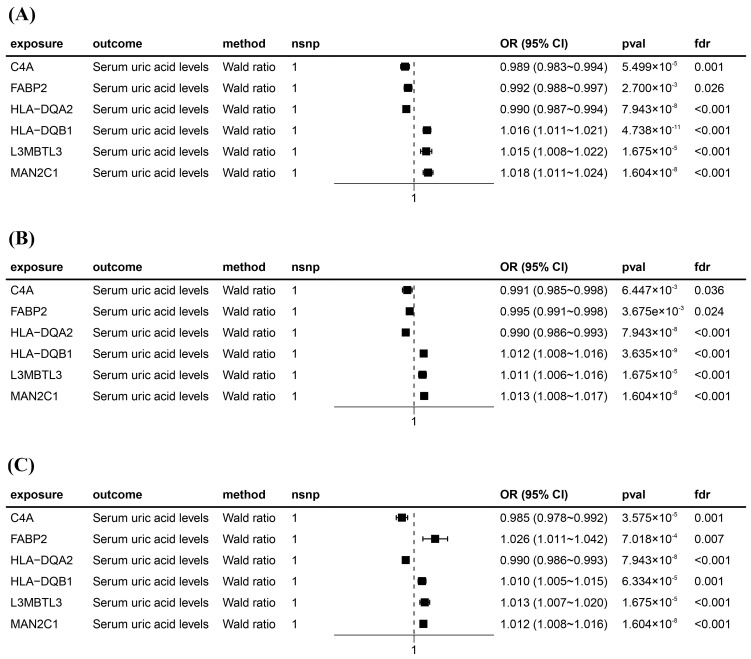
Forest plots for six significant Mendelian randomization (MR) result genes. (**A**) Forest plot for MR results between blood eQTLs and serum uric acid levels. (**B**) Forest plot for MR results between kidney eQTLs and serum uric acid levels. (**C**) Forest plot for MR results between intestine eQTLs and serum uric acid levels. The positions of the black squares (coordinates on the horizontal axis) represent the effect size; the horizontal line extending on either side of the black square indicates the confidence interval (usually 95% confidence interval) for the effect value.

After intersecting eQTLs from blood, kidney, and intestine tissues with druggable genes, respectively, we obtained 1696 gene symbols in blood eQTLs, 81 in kidney eQTLs, and 448 in intestine eQTLs. Using MR analysis, we identified 184 significant genes associated with serum UA levels in the blood, 14 in the kidneys, and 55 in the intestine following FDR adjustment (FDR < 0.05) (Figure 2). Among them, six genes—complement component 4A (*C4A*); fatty acid binding protein 2 (*FABP2*); major histocompatibility complex, class II, DQ alpha 2 (*HLA-DQA2*); major histocompatibility complex, class II, DQ beta 1 (*HLA-DQB1*); l3mbt-like 3 (*L3MBTL3*); and mannosidase alpha class 2C member 1 (*MAN2C1*)—were significant across all three tissues (Figure 3). Notably, the previously reported druggable gene ATP binding cassette subfamily G member 2 (*ABCG2*, a high-volume UA transporter) reached a significant level in the blood and exhibited the strongest association with UA levels, with an OR of 1.42 (Figure 2A). Detailed IV results for significant gene expressions and comprehensive MR findings are provided in Tables S4–S9.

Furthermore, we performed SMR and HEIDI tests on the significant genes screened by MR analysis in the blood, kidney, and intestinal tissues, using full summary-level data. The results demonstrated that 71, 6, and 19 genes in the blood, kidneys, and intestine, respectively, passed both the SMR test (FDR < 0.05) and the HEIDI test (*p* > 0.05) (Table S10). Eight genes—*FABP2*; major histocompatibility complex, class II, DQ alpha 1 (*HLA-DQA1*); *HLA-DQA2*; potassium two pore domain channel subfamily K member 17 (*KCNK17*); *L3MBTL3*; NADH:ubiquinone oxidoreductase subunit C2 (*NDUFC2*); *HLA-DQB1*; and adenosine A2b receptor (*ADORA2B*)—passed the SMR and HEIDI tests in at least two tissues of blood, kidney, and intestine. Among them, *L3MBTL3* met the significance criteria in all three tissues.

**Figure 4:**
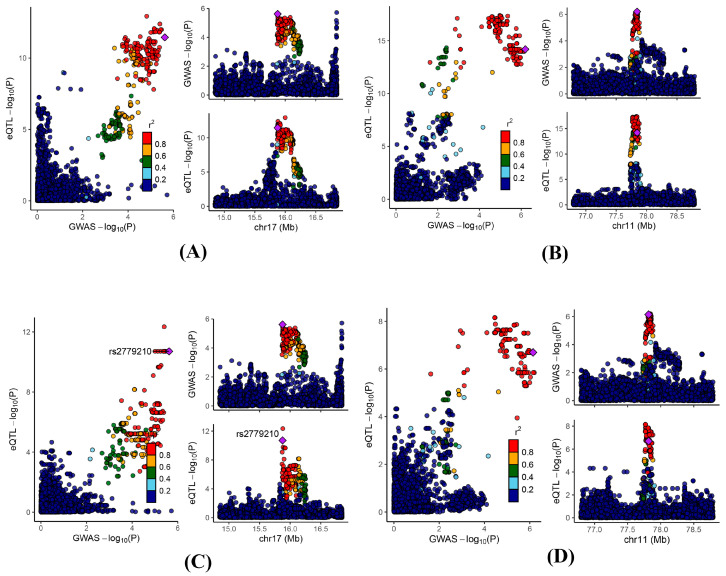
Regional genomic plots for conventional colocalization analysis. Colocalizations between serum uric acid levels and (**A**) blood *ADORA2B*; (**B**) blood *NDUFC2*; (**C**) kidney *ADORA2B*; and (**D**) intestine *NDUFC2*.

To further validate the observed findings, we performed a colocalization analysis on the significant genes identified through MR analysis. The results indicated that 25, 3, and 9 genes in the blood, kidney, and intestine, respectively, presented PPH4 values greater than 0.5 (Table S11). Notably, *ADORA2B* and *NDUFC2* exhibited significant colocalization in at least two of the examined tissues (blood, kidney, and intestine) (Figure 4).

Based on this cumulative evidence, we eventually identified 15, 2, and 7 genes in the blood, kidney, and intestine, respectively, that met all the criteria across the MR, SMR, and colocalization analyses. Among them, *ADORA2B* and *NDUFC2* passed all three tests in at least two tissues (blood, kidney, and intestine). The corresponding data are presented in Table 1.

### 3.3. Phenome-Wide Association Analysis

Given that *ADORA2B* and *NDUFC2* passed all three tests in at least two tissues, we conducted a phenome-wide association analysis of these two genes using the IEU OpenGWAS Project. The results demonstrated that ADORA2B (rs1683217) was strongly associated with serum UA levels, thereby corroborating the MR analysis findings. Additionally, *NDUFC2* (rs12937434) was primarily linked to immune and inflammatory indicators, including white blood cells, neutrophils, and lymphocytes. Furthermore, *NDUFC2* showed significant associations with serum UA levels, urea nitrogen levels, and gout. These findings further support the idea that *ADORA2B* and *NDUFC2* may be important genes that are highly correlated with serum UA levels without apparent adverse effects. The complete results are provided in Tables S12 and S13.

### 3.4. Protein–Protein Interaction and Enrichment Analysis

**Figure 5:**
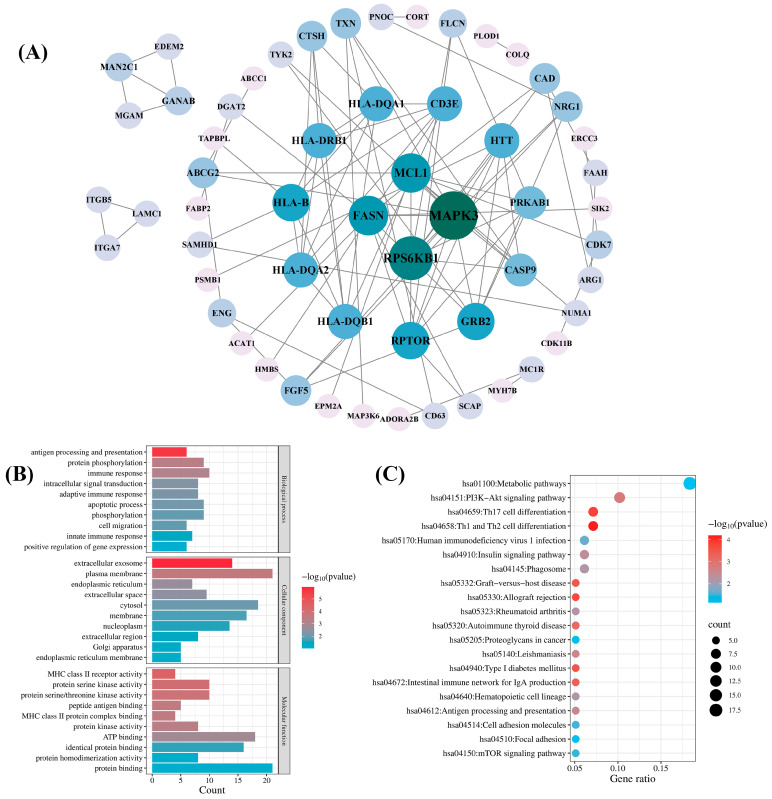
Network pharmacology analysis of the candidate druggable genes. (**A**) Protein–protein interaction (PPI) network analysis; (**B**) Gene Ontology (GO) biological function analysis; (**C**) Kyoto Encyclopedia of Genes and Genomes (KEGG) pathway analysis.

To comprehensively explore the biological functions of candidate druggable genes, we performed PPI, GO, and KEGG pathway analyses on a total of 98 genes from the blood, kidney, and intestinal tissues that met the MR significance criteria and either passed SMR or colocalization tests. A PPI network was constructed by inputting the 98 genes into the STRING database and it was then visualized with Cytoscape 3.9.1 software. As shown in Figure 5A, the network comprised 57 genes and 95 interactions. Among the 57 genes, mitogen-activated protein kinase 3 (*MAPK3*) and ribosomal protein S6 kinase B1 (*RPS6KB1*) exhibited higher connectivity, with degree values of 12 and 10, respectively. The remaining 41 genes showed no detectable protein interactions.

GO analysis of the 98 candidate genes revealed their predominant involvement in the immune response, protein phosphorylation, the apoptotic process, and phosphorylation in the BP category. In the CC category, the enriched terms included plasma membrane, cytosol, membrane, and extracellular exosome. In the MF category, the primary terms were protein binding, ATP binding, and identical protein binding (Figure 5B). KEGG pathway analysis revealed significant enrichment in the hsa01100 metabolic pathway and the hsa04151 PI3K-Akt signaling pathway (Figure 5C).

### 3.5. Candidate Drug Prediction

In the MR, SMR, and colocalization analyses, we identified a total of 22 genes in blood, kidney, and intestinal tissues that passed all three tests; accordingly, we performed drug predictions for these 22 significant druggable genes using the DSigDB database. The top 15 potential drugs were selected based on *p*-values. As shown in Table 2, chlorzoxazone (chlorzoxazone HL60 UP) and paclitaxel (paclitaxel CTD 00007144) emerged as the two most significant drugs. Chlorzoxazone interacted with fibroblast growth factor 5 (*FGF5*), cortistatin (*CORT*), opioid related nociceptin receptor 1 (*OPRL1*), neuregulin 1 (*NRG1*), and regulator of G protein signaling 12 (*RGS12*), whereas paclitaxel targeted *FGF5*, ATP binding cassette subfamily C member 1 (*ABCC1*), cyclin dependent kinase 7 (*CDK7*), integrin subunit beta 5 (*ITGB5*), and proteasome 20S subunit beta 1 (*PSMB1*). In addition, vinblastine (vinblastine CTD 00006986) was associated with the key druggable gene *NDUFC2*, and dipyridamole (dipyridamole BOSS) showed connectivity to the key druggable gene *ADORA2B*.

### 3.6. Molecular Docking Verification

**Figure 6:**
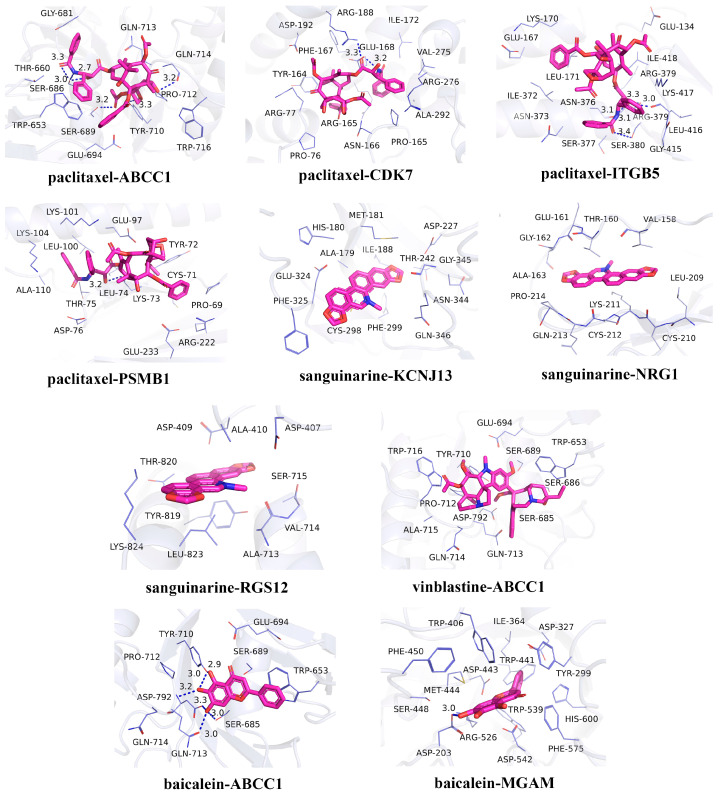
Molecular docking results for the candidate drugs and proteins with binding energies less than −5 kcal/mol.

In recent years, natural products have gained significant attention in the prevention and management of HUA due to their demonstrated therapeutic efficacy and favorable safety profiles [44]. In the candidate drug prediction analysis of this study, we identified 15 potential drugs, including four natural compounds. Given the therapeutic potential of natural products for HUA treatment, we selected these four natural compounds (paclitaxel, sanguinarine, vinblastine, and baicalein) along with their corresponding targets for molecular docking studies. The docking results (Table 3) revealed 13 significant drug–protein interactions, with 10 pairs demonstrating binding energies below -5 kcal/mol, indicating strong molecular affinity. The specific amino acid residues and hydrogen bond lengths are shown in Figure 6. Notably, the paclitaxel–CDK7 interaction exhibited the lowest binding energy (−7.8 kcal/mol), suggesting particularly strong binding characteristics.

## 4. Discussion

In this study, we employed an integrated approach combining MR, SMR, and colocalization methods to investigate the causal relationships between blood, kidney, and intestine eQTLs and serum UA level GWAS data, identifying 22 druggable genes that potentially influence serum UA levels. Phenome-wide association studies confirmed two key genes, *ADORA2B* and *NDUFC2*, as being significantly associated with serum UA levels while demonstrating favorable safety profiles. Subsequent PPI network and enrichment analyses of these candidate genes elucidated their biological functions and interaction mechanisms. Furthermore, drug prediction for the 22 druggable genes yielded potential therapeutic candidates for HUA treatment, with molecular docking studies confirming strong binding affinities between the selected drugs and their targets.

*ADORA2B* encodes an adenosine receptor belonging to the G protein-coupled receptor superfamily. Emerging evidence highlights ADORA2B as a promising therapeutic target in numerous pathological conditions, including inflammatory processes, immune responses, ischemia–reperfusion injury, fibrosis, and diabetes mellitus [45]. Given its role as an adenosine receptor, ADORA2B is intrinsically linked to UA metabolism, which may serve as an entry point to reduce UA production because adenosine is a precursor in UA synthesis. Previous studies by Eckle et al. reported that ADORA2B upregulated hypoxia-inducible factor 1 alpha expression through stabilization of the circadian rhythm protein period 2 [46]. Notably, hypoxia-inducible factor 1 alpha plays a crucial role in the synthesis of xanthine oxidase, the key enzyme responsible for catalyzing xanthine’s conversion into UA. The xanthine oxidase regulatory domain contains hypoxia-responsive elements, and its expression is mediated by activator protein-1, whose accumulation represents an energy-intensive process dependent on hypoxia-inducible factor 1 [47,48]. In addition, Shen et al. reported that ADORA2B inhibited the PI3K/Akt signaling pathway; specifically, the knockdown of ADORA2B prominently increased the phosphorylation levels of PI3K and Akt in chondrocytes [49]. Interestingly, several Chinese herbal compounds (e.g., naringenin and coptisine) have shown UA-lowering effects through PI3K/Akt pathway inhibition [50]. Notably, our enrichment analysis also identified the PI3K/Akt pathway, suggesting that ADORA2B’s potential regulatory role in this pathway warrants further investigation.

*NDUFC2* encodes an essential subunit of mitochondrial complex I, serving as an accessory component of the mitochondrial membrane respiratory chain NADH dehydrogenase. This subunit plays a critical role in the proper assembly and function of the complex [51]. Notably, NDUFC2 downregulation disrupts complex I assembly, leading to decreased complex I activity, impaired adenosine triphosphate (ATP) synthesis, and elevated reactive oxygen species (ROS) production [52]. Previous studies have shown that the process by which xanthine oxidase catalyzes the metabolism of xanthine and hypoxanthine to UA is accompanied by ROS generation [53]. Moreover, UA exhibits dual redox properties, functioning as both an antioxidant and a pro-oxidant that activates NADPH oxidase to generate ROS [54]. In addition, dysregulation of NDUFC2 has been reported to impair mitochondrial ROS clearance and contribute to insulin resistance. Functionally, the knockdown of NDUFC2 results in a significant reduction in glucose uptake and the inhibition of gene transcription related to the insulin and glucose metabolism pathways [55]. Accumulating evidence indicates a strong association between HUA and insulin resistance. On the one hand, some studies have suggested that HUA is an independent risk factor for insulin resistance, with elevated UA levels preceding insulin resistance development [56,57]. On the other hand, some recent studies have shown that reducing insulin resistance may lower serum UA levels and reduce gout risk [58,59]. These findings implicate NDUFC2 in UA regulation through oxidative stress and insulin resistance pathways.

To identify potential therapeutic agents targeting candidate druggable genes, we employed the DSigDB database for drug prediction. Notably, multiple testing correction was omitted to obtain a sufficient number of predicted drugs; however, this may increase the risk of false-positive associations, which warrants cautious interpretation and further validation. Molecular docking simulations revealed strong binding affinities between the identified drugs and target genes. Among the top 15 predicted compounds, baicalein, sanguinarine, dipyridamole, and bucladesine have been previously reported to modulate HUA. Baicalin, a natural flavonoid extract isolated from the Chinese herb *Scutellariae radix*, has shown multiple anti-HUA effects. Meng et al. demonstrated that baicalein effectively lowered serum UA levels in HUA mice by inhibiting xanthine oxidase activity while ameliorating renal fibrosis and epithelial–mesenchymal transition, thereby exhibiting renal-protective properties [60]. Similarly, Chen et al. reported that baicalin inhibited the UA transporters solute carrier family 2 member 9 (GLUT9) and uric acid transporter 1 (URAT1) in a non-competitive dose-dependent manner, and inhibited xanthine oxidase activity to reduce UA levels in HUA mice [61]. Furthermore, Liu et al. elucidated its mechanism of action, showing that baicalein attenuates hyperuricemic nephropathy through PI3K/AKT/NF-κB pathway inhibition, thereby reducing renal inflammation and apoptosis [62]. In addition, Zeng et al. identified baicalein as a key active component in Simiao pills, which significantly reduced serum UA levels, improved renal injury caused by HUA, and suppressed the NF-κB/NLRP3/IL-1β signaling pathway [63]. Sanguinarine, a bioactive compound derived from *Macleaya cordata*, has been shown to improve growth performance while reducing UA and urea nitrogen levels in broilers by regulating the gut microbiome [64]. Dipyridamole is an antiplatelet agent that has been reported to exhibit multiple beneficial effects, including reducing blood UA, urea nitrogen, and potassium levels; ameliorating oxidative stress; and protecting against ischemia–reperfusion-induced acute kidney injury in rats [65]. Notably, dipyridamole may also mitigate the purine overproduction associated with HUA and gout [66]. Bucladesine sodium exerts diverse physiological effects, such as increasing insulin secretion, increasing urine output, and increasing the blood renin concentration. Yamamoto et al. reported its ability to modulate renal hypoxanthine transport, resulting in increased urinary excretion of hypoxanthine and UA in healthy subjects [67]. Although current evidence is primarily derived from preclinical studies, these findings suggest the potential relevance of the compounds for HUA treatment. Further clinical investigations are warranted to validate their efficacy and safety in human patients.

The major strength of this study lies in our utilization of the most comprehensive publicly available GWAS data on serum UA levels, providing robust genetic evidence through the integrated application of MR, SMR, and colocalization analyses for identifying druggable targets for HUA. Notably, our eQTL selection strategy incorporated not only blood-derived data but also kidney and intestinal eQTL datasets, recognizing that UA excretion involves both renal and intestinal pathways. This comprehensive approach provided more complete genetic expression information for target identification. Additional evidence from PPI analysis, enrichment analysis, and phenome-wide association analysis provided insights into the functional characteristics, safety, and potential therapeutic mechanisms of potential drug targets, thereby facilitating target prioritization. Furthermore, our drug prediction and molecular docking studies identified promising therapeutic candidates for HUA, with previously reported UA-lowering effects of these compounds further validating the potential of our candidate targets.

Several limitations should be acknowledged. First, the lack of comprehensive pQTL data for blood, kidney, and intestine tissues in public databases constrained our ability to perform exhaustive pQTL analyses of potential targets. The collection and analysis of pQTL datasets should be considered in the future, which will provide a more detailed study on the target screening of HUA. Second, the limited number of eQTL instrumental variables (typically ≤ 3 SNPs per gene) may compromise the reliability of MR results. Third, while our computational drug prediction and molecular docking approaches identified potential therapeutic agents, these in silico findings require experimental validation to confirm clinical efficacy. Most importantly, as MR analysis establishes associations rather than causal relationships, our findings necessitate verification through both basic research and clinical trials to establish therapeutic relevance.

## 5. Conclusions

In this study, we employed MR, SMR, and colocalization analyses to systematically identify 22 potential therapeutic targets for HUA treatment and subsequently screened 15 candidate drugs targeting these genes. These findings provide promising leads for the discovery of novel therapeutic targets and candidate drugs for HUA. Further experimental validation and clinical investigations are required to assess the therapeutic potential and clinical efficacy of these targets and drug candidates.

## Figures and Tables

**Table 1:** Summary results from Mendelian randomization (MR), summary-data-based MR (SMR), and colocalization for 22 important candidate druggable genes.

Gene Name	Tissue	MR			SMR		Colocalization
		OR (95% CI)	*p*-Value	FDR	*p*-HEIDI	FDR	PPH4
*ABCC1*	blood	0.968	0.002	0.020	0.736	0.036	0.589
*ADORA2B*	blood	1.035	<0.001	<0.001	0.091	0.005	0.865
*CAMLG*	blood	1.027	0.001	0.014	0.842	0.030	0.650
*CD180*	blood	0.972	<0.001	0.003	0.316	0.010	0.856
*CDK7*	blood	0.912	<0.001	<0.001	0.346	0.001	0.781
*CORT*	blood	1.028	<0.001	0.001	0.683	0.016	0.644
*ITGB5*	blood	0.939	<0.001	<0.001	0.409	0.008	0.612
*MGAM*	blood	0.944	0.001	0.008	0.919	0.026	0.736
*MUCL1*	blood	0.977	0.001	0.011	0.093	0.040	0.639
*NDUFC2*	blood	0.951	<0.001	<0.001	0.496	0.003	0.589
*NRG1*	blood	0.966	<0.001	<0.001	0.531	<0.001	0.999
*OPRL1*	blood	1.038	<0.001	<0.001	0.233	0.001	0.594
*PSMB1*	blood	0.958	0.001	0.015	0.092	0.031	0.527
*RGS12*	blood	0.957	0.001	0.013	0.151	0.046	0.620
*RPTOR*	blood	1.040	0.001	0.009	0.349	0.017	0.663
*ADORA2B*	kidney	1.008	<0.001	<0.001	0.708	0.002	0.904
*FGF5*	kidney	0.973	<0.001	<0.001	0.112	<0.001	0.938
*DHX36*	intestine	0.961	<0.001	<0.001	0.127	0.002	0.826
*FABP2*	intestine	1.026	0.001	0.007	0.150	0.038	0.501
*FLCN*	intestine	0.991	<0.001	<0.001	0.452	0.003	0.880
*HLA-DQA1*	intestine	0.975	<0.001	<0.001	0.972	0.008	0.542
*KCNJ13*	intestine	1.026	0.001	0.006	0.665	0.038	0.799
*NDUFC2*	intestine	0.960	<0.001	0.001	0.265	0.017	0.764
*SLC5A9*	intestine	1.042	<0.001	0.001	0.351	0.014	0.906

MR, Mendelian randomization; SMR, summary-based Mendelian randomization; OR, odds ratio; FDR, false discovery rate; HEIDI, heterogeneity in dependent instruments; PPH4, posterior probability of hypothesis 4.

**Table 2:** The candidate drugs predicted by the DSigDB database.

Drug Name	*p*-Value	Odds Ratio	Genes
chlorzoxazone HL60 UP	<0.001	12.763	*FGF5;CORT;OPRL1;NRG1;RGS12*
paclitaxel CTD 00007144	<0.001	11.747	*FGF5;ABCC1;CDK7;ITGB5;PSMB1*
sulfamonomethoxine HL60 UP	<0.001	13.480	*CORT;KCNJ13;OPRL1;NRG1*
idarubicin CTD 00007058	<0.001	71.250	*ABCC1;RGS12*
cefoxitin HL60 UP	0.001	18.618	*CORT;KCNJ13;NRG1*
iopromide HL60 UP	0.001	16.444	*CORT;KCNJ13;NRG1*
sanguinarine MCF7 UP	0.001	16.186	*KCNJ13;NRG1;RGS12*
vinblastine CTD 00006986	0.002	13.924	*ABCC1;PSMB1;NDUFC2*
bucladesine HL60 UP	0.002	13.557	*CORT;KCNJ13;NRG1*
dipyridamole BOSS	0.002	33.197	*ADORA2B;RGS12*
enoxaparin CTD 00006081	0.002	32.123	*FGF5;ABCC1*
baicalein CTD 00000302	0.003	28.440	*MGAM;ABCC1*
disodium selenite CTD 00007229	0.003	5.117	*RPTOR;CDK7;CAMLG;KCNJ13;* *PSMB1;DHX36*
lamivudine CTD 00007258	0.003	27.647	*ABCC1;CDK7*
alimemazine HL60 UP	0.004	25.189	*KCNJ13;NRG1*

**Table 3:** Docking results of the candidate drugs and proteins.

Compound	Target	Affinity (Kcal/mol)
paclitaxel	ABCC1	−6.6
	CDK7	−7.8
	FGF5	−4.2
	ITGB5	−6.4
	PSMB1	−5.5
sanguinarine	KCNJ13	−7.6
	NRG1	−6.8
	RGS12	−7.4
vinblastine	ABCC1	−5.7
	NDUFC2	−4.6
	PSMB1	−4.6
baicalein	ABCC1	−6.0
	MGAM	−7.6

## Data Availability

All code and data have been uploaded to GitHub (https://github.com/jiumeng-bit/MR-for-hyperuricemia, accessed on 24 March 2025).

## Supplementary Materials

The following supporting information can be downloaded at: https://www.mdpi.com/article/10.3390/biomedicines13051022/s1. Table S1: The potential druggable genes in DGIdb database. More details are shown in DGIdb; Table S2: The druggable genes from a review by Finan C et al.; Table S3: The final list of potential druggable genes; Table S4: The instrumental variants and F statistics for blood eQTLs; Table S5: The instrumental variants and F statistics for kidney eQTLs; Table S6: The instrumental variants and F statistics for intestine eQTLs; Table S7: MR results of blood eQTLs and serum UA levels; Table S8: MR results of kidney eQTLs and serum UA levels; Table S9: MR results of intestine eQTLs and serum UA levels; Table S10: The significant results of SMR analysis; Table S11: The significant results of colocalization analysis; Table S12: The phenome-wide association analysis results of *ADORA2B*; Table S13: The phenome-wide association analysis results of *NDUFC2*. References [68,69] are cited in the Supplementary Materials.
