# Supplementary material for: Identification of Therapeutic Targets for Hyperuricemia: Systematic Genome-Wide Mendelian Randomization and Colocalization Analysis
Source: Biomedicines. 2025 Apr 23;13(5):1022. doi: 10.3390/biomedicines13051022 (PMC12109542; doi:10.3390/biomedicines13051022)
Supplement: Supplementary file 1 [file biomedicines-13-01022-s001.zip › STROBE-MR-checklist.pdf]

STROBE-MR checklist of recommended items to address in reports of Mendelian randomization studies[1 2]

Item No.	Section	Checklist item	Page No.	Relevant text from manuscript
1	**TITLE and ABSTRACT**	Indicate Mendelian randomization (MR) as the study's design in the title and/or the abstract if that is a main purpose of the study	1	Mendelian randomization
	**INTRODUCTION**			
2	**Background**	Explain the scientific background and rationale for the reported study. What is the exposure? Is a potential causal relationship between exposure and outcome plausible? Justify why MR is a helpful method to address the study question	2	Therefore, there is a pressing need to explore the underlying pathogenesis of HUA and identify novel therapeutic targets.<br>Mendelian randomization (MR) is a method that uses genetic variation as an instru-mental variable to infer causal relationships between exposure and outcome and is often regarded as a natural randomized controlled trial [16]. As an emerging method for causal inference, MR has demonstrated remarkable strengths in the field of drug target identification by integrating aggregated data from disease GWAS and expression quan-titative trait loci (eQTL) studies. The eQTLs identified within the genomic domains of pharmacologically actionable genes are often regarded as proxies, given that the expression levels of these genes can be interpreted as a form of lifelong exposure [19]. Hence, MR-based screening of target proteins has robust natural advantages and genetic substantiation.
3	**Objectives**	State specific objectives clearly, including pre-specified causal hypotheses (if any). State that MR is a method that, under specific assumptions, intends to estimate causal effects	2	In this study, we performed a systematic druggable genome-wide MR analysis to identify therapeutic targets for HUA. First, we obtained data on druggable genes and conducted a meticulous screening process, focusing on genes associated with blood eQTLs, kidney eQTLs, and intestine eQTLs. These selected genes were then subjected to two-sample MR analysis with genome-wide association studies (GWAS) data of serum UA levels to identify genes strongly associated with HUA.
	**METHODS**			

4	**Study design and data sources**	Present key elements of the study design early in the article. Consider including a table listing sources of data for all phases of the study. For each data source contributing to the analysis, describe the following:		
	a)	Setting: Describe the study design and the underlying population, if possible. Describe the setting, locations, and relevant dates, including periods of recruitment, exposure, follow-up, and data collection, when available.	3	Figure 1. Overview of the study design
	b)	Participants: Give the eligibility criteria, and the sources and methods of selection of participants. Report the sample size, and whether any power or sample size calculations were carried out prior to the main analysis	3	The study utilized GWAS summary statistics derived from a large-scale cohort.
	c)	Describe measurement, quality control and selection of genetic variants	3	We selected all statistically significant eQTLs ($p < 1\times10^{-8}$).
	d)	For each exposure, outcome, and other relevant variables, describe methods of assessment and diagnostic criteria for diseases	3	The outcome data for HUA represented by serum UA levels (GWAS ID: ebi-a-GCST90018977).
	e)	Provide details of ethics committee approval and participant informed consent, if relevant	3	The outcome data for HUA represented by serum UA levels (GWAS ID: ebi-a-GCST90018977).
5	**Assumptions**	Explicitly state the three core IV assumptions for the main analysis (relevance, independence and exclusion restriction) as well assumptions for any additional or sensitivity analysis	4	The selection of eQTLs adhered to the following criteria: (1) Only eQTLs demonstrating a significant association with the exposure ($P < 1\times10^{-8}$) were included. (2) To minimize linkage disequilibrium (LD) bias, eQTLs were required to be independent, defined by r2 threshold of < 0.001 using European samples from the 1000 Genomes Project and a minimum genetic distance of 10,000 kb. (3) eQTLs exhibiting associations with potential confounders or outcomes were excluded to ensure specificity.
6	**Statistical methods: main analysis**	Describe statistical methods and statistics used		
	a)	Describe how quantitative variables were handled in the analyses (i.e., scale, units, model)	4	The selection of eQTLs adhered to the following criteria: (1) Only eQTLs demonstrating a significant association with the exposure ($P < 1\times10^{-8}$) were included. (2) To minimize linkage disequilibrium (LD) bias, eQTLs were required to be independent, defined by r2 threshold of < 0.001 using European samples from the 1000 Genomes Project and a minimum genetic distance of 10,000 kb. (3) eQTLs exhibiting

					associations with potential confounders or outcomes were excluded to ensure specificity.
		b)	Describe how genetic variants were handled in the analyses and, if applicable, how their weights were selected	4	The strength of IVs was estimated using the F statistic and a threshold of F > 10 was selected to exclude weak instruments.
		c)	Describe the MR estimator (e.g. two-stage least squares, Wald ratio) and related statistics. Detail the included covariates and, in case of two-sample MR, whether the same covariate set was used for adjustment in the two samples	4	The Wald ratio method was used to perform MR analysis when only one SNP was available for analysis, whereas the inverse-variance weighted (IVW) method with random effects was used to conduct MR estimation when multiple SNPs were available.
		d)	Explain how missing data were addressed	-	-
		e)	If applicable, indicate how multiple testing was addressed	4	Statistical significance was defined as false discovery rate (FDR) < 0.05.
7	**Assessment of assumptions**		Describe any methods or prior knowledge used to assess the assumptions or justify their validity	-	-
8	**Sensitivity analyses and additional analyses**		Describe any sensitivity analyses or additional analyses performed (e.g. comparison of effect estimates from different approaches, independent replication, bias analytic techniques, validation of instruments, simulations)	4	Cochran's Q test was used to test for heterogeneity among the individual causal effects of SNP, while MR-Egger was performed to consider horizontal pleiotropy.
9	**Software and pre-registration**				
		a)	Name statistical software and package(s), including version and settings used		The "TwoSampleMR" package (version 0.6.6) was employed to perform MR analysis using R software.
		b)	State whether the study protocol and details were pre-registered (as well as when and where)	-	-
	**RESULTS**				
10	**Descriptive data**				
		a)	Report the numbers of individuals at each stage of included studies and reasons for exclusion. Consider use of a flow diagram	3	Figure 1. Overview of the study design
		b)	Report summary statistics for phenotypic exposure(s), outcome(s), and other relevant variables (e.g. means, SDs, proportions)	3	The outcome data for HUA represented by serum UA levels (GWAS ID: ebi-a-GCST90018977), comprised genetic data from 343,836

					participants of European ancestry with 19,041,286 SNPs.
		c)	If the data sources include meta-analyses of previous studies, provide the assessments of heterogeneity across these studies	-	-
		d)	For two-sample MR:<br>i. Provide justification of the similarity of the genetic variant-exposure associations between the exposure and outcome samples<br>ii. Provide information on the number of individuals who overlap between the exposure and outcome studies	-	-
11	**Main results**				
		a)	Report the associations between genetic variant and exposure, and between genetic variant and outcome, preferably on an interpretable scale	6	After intersecting eQTLs from blood, kidney, and intestine tissues with druggable genes respectively, we obtained 1,696 gene symbols in blood eQTLs, 81 in kidney eQTLs, and 448 in intestine eQTLs.
		b)	Report MR estimates of the relationship between exposure and outcome, and the measures of uncertainty from the MR analysis, on an interpretable scale, such as odds ratio or relative risk per SD difference	6	Using MR analysis, we identified 184 significant genes associated with serum UA levels in blood, 14 in kidney, and 55 in intestine following FDR adjustment (FDR < 0.05).
		c)	If relevant, consider translating estimates of relative risk into absolute risk for a meaningful time period	-	-
		d)	Consider plots to visualize results (e.g. forest plot, scatterplot of associations between genetic variants and outcome versus between genetic variants and exposure)	7, 8	Figure 2. Volcano plots for Mendelian randomization (MR) results.<br>Figure 3. Forest plots for six significant Mendelian randomization (MR) result genes.
12	**Assessment of assumptions**				
		a)	Report the assessment of the validity of the assumptions	6	Detailed IV results for significant gene expressions and comprehensive MR findings are provided in Tables S4-S9.
		b)	Report any additional statistics (e.g., assessments of heterogeneity across genetic variants, such as I^2, Q statistic or E-value)	6	Detailed IV results for significant gene expressions and comprehensive MR findings are provided in Tables S4-S9.
13	**Sensitivity analyses and**				

	**additional analyses**				
		a)	Report any sensitivity analyses to assess the robustness of the main results to violations of the assumptions	6	Detailed IV results for significant gene expressions and comprehensive MR findings are provided in Tables S4-S9.
		b)	Report results from other sensitivity analyses or additional analyses	6, 8	Furthermore, we performed SMR and HEIDI tests on the significant genes screened by MR analysis in blood, kidney, and intestinal tissues, using full summary-level data.<br>To further validate the observed findings, we performed a colocalization analysis on the significant genes identified through MR analysis.
		c)	Report any assessment of direction of causal relationship (e.g., bidirectional MR)	6	Detailed IV results for significant gene expressions and comprehensive MR findings are provided in Tables S4-S9.
		d)	When relevant, report and compare with estimates from non-MR analyses	6, 8	Furthermore, we performed SMR and HEIDI tests on the significant genes screened by MR analysis in blood, kidney, and intestinal tissues, using full summary-level data.<br>To further validate the observed findings, we performed a colocalization analysis on the significant genes identified through MR analysis.
		e)	Consider additional plots to visualize results (e.g., leave-one-out analyses)	9	Figure 4. Regional genomic plots for conventional colocalization analysis.<br>Table 1. Summary results from Mendelian randomization (MR), summary-data-based MR (SMR), and colocalization for 22 important candidate druggable genes.
	**DISCUSSION**				
14	**Key results**		Summarize key results with reference to study objectives	13	In this study, we employed an integrated approach combining MR, SMR, and colocalization methods to investigated the causal relationships between blood, kidney, and intestine eQTLs and serum UA level GWAS data, identifying 22 druggable genes that potentially influencing serum UA levels. Phenome-wide association studies confirmed two key genes, ADORA2B and NDUFC2, as being significantly

					associated with serum UA levels while demonstrating favorable safety profiles.
15	**Limitations**		Discuss limitations of the study, taking into account the validity of the IV assumptions, other sources of potential bias, and imprecision. Discuss both direction and magnitude of any potential bias and any efforts to address them	15	Several limitations should be acknowledged. First, the lack of comprehensive pQTL data for blood, kidney, and intestine tissues in public databases constrained our ability to perform exhaustive pQTL analyses of potential targets. The collection and analysis of pQTL datasets should be considered in the future, which will provide a more detailed study on the target screening of HUA. Second……
16	**Interpretation**				
		a)	Meaning: Give a cautious overall interpretation of results in the context of their limitations and in comparison with other studies	15	These findings provide promising leads for the discovery of novel therapeutic targets and candidate drugs for HUA. Further experimental validation and clinical investigations are required to assess the therapeutic potential and clinical efficacy of these targets and drug candidates.
		b)	Mechanism: Discuss underlying biological mechanisms that could drive a potential causal relationship between the investigated exposure and the outcome, and whether the gene-environment equivalence assumption is reasonable. Use causal language carefully, clarifying that IV estimates may provide causal effects only under certain assumptions	14	Given its role as an adenosine receptor, ADORA2B is intrinsically linked to UA metabolism, which may serve as an entry point to reduce UA production since adenosine is a precursor in UA synthesis.<br>These findings implicate NDUFC2 in UA regulation through oxidative stress and insulin resistance pathways.
		c)	Clinical relevance: Discuss whether the results have clinical or public policy relevance, and to what extent they inform effect sizes of possible interventions	13	drug prediction for the 22 druggable genes yielded potential therapeutic candidates for HUA treatment, with molecular docking studies confirming strong binding affinities between selected drugs and their targets.
17	**Generalizability**		Discuss the generalizability of the study results (a) to other populations, (b) across other exposure periods/timings, and (c) across other levels of exposure	15	Most importantly, as MR analysis establishes associations rather than causal relationships, our findings necessitate verification through both basic research and clinical trials to establish therapeutic relevance.
	**OTHER INFORMATION**				

18	**Funding**	Describe sources of funding and the role of funders in the present study and, if applicable, sources of funding for the databases and original study or studies on which the present study is based	17	This work was supported by the National Natural Science Foundation of China (No. 82304802).
19	**Data and data sharing**	Provide the data used to perform all analyses or report where and how the data can be accessed, and reference these sources in the article. Provide the statistical code needed to reproduce the results in the article, or report whether the code is publicly accessible and if so, where	17	All code and data have been uploaded to GitHub (https://github.com/jiumeng-bit/MR-for-hyperuricemia).
20	**Conflicts of Interest**	All authors should declare all potential conflicts of interest	17	The authors declare that they have no competing interests.

This checklist is copyrighted by the Equator Network under the Creative Commons Attribution 3.0 Unported (CC BY 3.0) license.

1. Skrivankova VW, Richmond RC, Woolf BAR, Yarmolinsky J, Davies NM, Swanson SA, et al. Strengthening the Reporting of Observational Studies in Epidemiology using Mendelian Randomization (STROBE-MR) Statement. JAMA. 2021;under review.

2. Skrivankova VW, Richmond RC, Woolf BAR, Davies NM, Swanson SA, VanderWeele TJ, et al. Strengthening the Reporting of Observational Studies in Epidemiology using Mendelian Randomisation (STROBE-MR): Explanation and Elaboration. BMJ. 2021;375:n2233.
